# Reducing opioid utilization after appendectomy: A lesson in implementation of a multidisciplinary quality improvement project^☆^

**DOI:** 10.1016/j.sopen.2019.08.001

**Published:** 2019-10-22

**Authors:** Kimberly K Somers, Ruchi Amin, Kathleen M Leack, Melissa Lingongo, Marjorie J Arca, David M Gourlay

**Affiliations:** Children's Hospital of Wisconsin and Division of Pediatric Surgery, Department of Surgery, Medical College of Wisconsin, Milwaukee, WI

## Abstract

**Background:**

Perioperative care after appendectomy may be the first exposure to opioids for many children. A quality improvement project was implemented to assess current practice of prescribing pain medications after a laparoscopic appendectomy to decrease unnecessary opioid use via simple, targeted steps.

**Methods:**

Three measures were implemented in patients undergoing laparoscopic appendectomy for acute appendicitis: (1) ice packs to incision in postanesthesia care unit, (2) standard pain scores within 30 minutes of admission to ward postoperatively, and (3) standardized postoperative order set minimizing opioid utilization and limited number of opioids prescribed at discharge. Pre- and postimplementation data were compared with the primary outcome variable: opioid utilization during the postoperative period.

**Results:**

There were no statistically significant differences in age or gender between the 814 preimplementation and 263 postimplementation patients. Postimplementation compliance is 66.9% for icepacks, 88% for pain scores, and 94.7% for postoperative order set. There were statistically significant decreases in intravenous and enteral opioids administered, number of opioid doses prescribed at discharge, and patients discharged with an opioid prescription.

**Conclusion:**

By using a multidisciplinary assessment of current state, culture, and management of parental, patient, and nursing expectations, our institution was able to reduce overall opioid consumption.

## Background/Available Knowledge

There is a current opioid crisis in the United States. Kane et al noted that there is an increase in hospitalizations and critical care admissions for ingestions in the pediatric population, and the death rate from opioid medications doubled since 2000 [1]. Prescription opioid exposure in young children and adolescents continues to emerge as the leading source of poisoning in children up to age 6 years followed by teenagers despite efforts like prescription drug monitoring programs, and community and governmental prevention programs [1,2]. Because appendicitis is the most common urgent operation in children with approximately 70,000 pediatric appendectomies performed annually, it stands to reason that many children may have their index exposure to opioids around the time of their appendectomies [3]. The average cost of a hospitalization for appendicitis is $9000, which accounts for 30% of the cumulative cost of all pediatric general surgical conditions combined [4]. The financial burden of this disease is further complicated by return visits to the emergency department and outpatient clinic, as well as hospital readmissions for inadequate pain control and narcotic-related complaints. There have been prospective studies performed to compare non-narcotic outpatient oral analgesia to narcotic-based therapy. These have suggested that non-narcotic therapy has equivalent outcomes and results in higher parental satisfaction for pediatric patients with acute appendicitis [5]. Despite these data, there remains a large variation in practice regarding postoperative pain management for these children. The national surgical quality improvement program provides nationally validated outcomes that are risk adjusted to establish benchmarks with similar organizations. Once identified as an outlier, organizations can complete in-depth assessments of practice to help determine interventions. To better understand the large variation in practice, national benchmarks need to be established and in-depth assessments of current state completed. By completing an in-depth assessment of current state pain management practices, organizations can determine root causes which are contributing to the opioid crisis.

## Rationale

Guidelines established by the American Pain Society recommend the concomitant use of acetaminophen and nonsteroidal inflammatory drug (NSAID) pain medications in the postoperative pain management of pediatric patients [6]. In a meta-analysis, Michelet et al found that coadministration of NSAIDs and opioids decreased opioid use, decreased opioid adverse effects, and provided better pain control [7]. In addition, there is evidence suggesting that the differing mechanisms of action of acetaminophen and/or NSAIDs are more effective than either drug alone for the treatment of postoperative pain [[8], [9], [10], [11], [12]]. Based on these studies, we maximized the use of acetaminophen and ibuprofen as the primary analgesic agents in our protocols, using oxycodone for breakthrough pain control only.

## Specific Aims

By studying past and current practice and defining a process for prescribing pain medications after laparoscopic appendectomy, we can provide appropriate pain control and decrease unnecessary opioid use. The aim of this study was to determine whether a systematic protocol for postoperative pain reduction would reduce opioid utilization. We also wanted to monitor the incidence of postdischarge returns for pain and opioid-related problems such as constipation in this patient population.

## Methods

### Context

Children's Hospital of Wisconsin (CHW) is a 298-bed tertiary American College of Surgeons–verified Level I Children's Surgical Center affiliated with the Medical College of Wisconsin. Approximately 350 appendectomies are performed at CHW annually with 250 for acute appendicitis. A single group of pediatric surgeons and providers cared for these patients. Once developed, the entire group was engaged in the implementation of the quality improvement (QI) project. The number of acute appendectomies performed annually did not vary significantly before and after implementation of the interventions.

Data were obtained from the Appendectomy Pediatric Prospective Index, a database maintained by the Division of Pediatric Surgery that uses a REDCap [13] platform and contains all the patients who have undergone appendectomy at CHW since 2008. Demographic variables as well as preoperative, intraoperative, and postoperative outcomes are recorded. All postoperative data are reviewed and entered by a clinical provider. The database is maintained in a password-protected secure server with all exports deidentified to protect individual patient identity.

Institutional review board approval was obtained for collection and analysis of retrospective data used to inform the practice initiatives. The QI process and associated prospective data were deemed “Not Human Subjects Research” by the institutional review board.

### Process

The selection and implementation of quality interventions were based on a hands-on, project-based quality improvement training course developed at CHW based on Plan–Do–Study–Act (PDSA) methodology [14]. The surgical team was interested in decreasing postoperative in-hospital and discharge opioid utilization. To initiate the process, the team characterized existing narcotic and non-narcotic pain strategies for acute appendicitis by determining the total number of doses of parenteral and enteral opioids, nonpharmacologic interventions (ice packs), and doses of nonopioid medications. To ensure that we were not having unintentional consequences to our new protocol changes, we monitored for returns to the health system for ineffective pain control.

The QI team defined the problem by looking at institutional and national data (national surgical quality improvement program). Locally, there was a high incidence of unplanned returns to the medical system for pain and constipation. Based on these findings, the team defined its aims: (1) to reduce opioid use after appendectomy for acute appendicitis and (2) decrease unplanned returns to the system (emergency department, clinics, and hospital readmissions).

Data on patients from 2013 to 2016 were obtained. Hospital readmissions, emergency room, and clinic visits were plotted on run charts (P Chart) to assess the number of patients successfully discharged before a return to system occurred for pain or constipation. Prescriptions were evaluated for total doses prescribed at discharge and were validated in the Wisconsin Enhanced Prescription Drug Monitoring Program to determine if the prescription was filled. All prescriptions filled in the state of Wisconsin that are submitted by pharmacies and other dispensers are captured in this registry. The baseline data were used to inform the global aim: create an efficient and effective care process to reduce the incidence of postdischarge returns for pain and constipation, while decreasing opioid use in pediatric appendectomy patients, and the presence of opioids in the community.

**Fig 1:**
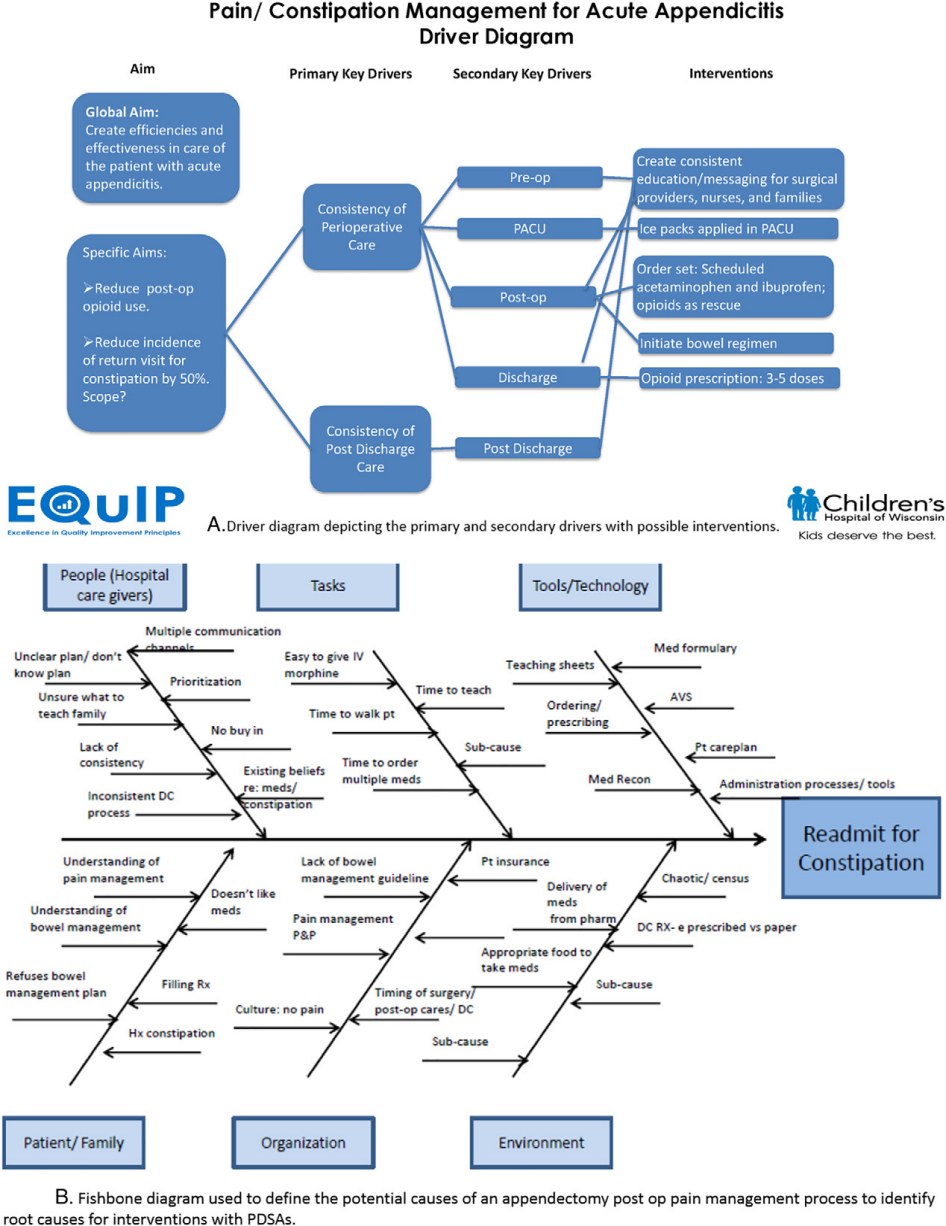
Tools utilized by the QI team to plan and implement QI project. A, Driver diagram depicting the primary and secondary drivers with possible interventions. B, Fishbone diagram used to define the potential causes of an appendectomy postoperative pain management process to identify root causes for interventions with PDSAs.

Once the team was assembled, meetings were held every 2 weeks. Each meeting had ground rules established, assessment tools used, and homework assigned. The first project was for the team leaders to create a driver diagram of the aim, as well as primary and secondary drivers (Fig 1, *A*). Once these were defined, the potential interventions were added. The aim and primary drivers were concrete: reduce opioid use during admission and at discharge, use primarily nonopioid analgesia medication, and obtain a 50% reduction in returns to the system for pain and constipation.

Analysis of current state included the use of a fish bone diagram (Fig 1, *B*) which occurred over 3 sessions of 1.5 hours each. Complex in-depth discussions led to multiple underlying causes for returns. Once the current state was defined, an impact/effort assessment tool [15] for rank ordering was completed by the team to determine the first PDSA with a focus on the potential for high return and ease of implementation. Based on the impact/effort assessment, the team limited the scope, as the fish bone demonstrated varied causes and numerous opportunities for intervention. Limiting the scope allowed for better team dynamics and engagement.

### Interventions

A retrospective review of 608 patients with acute appendicitis in our institution evaluating nonpharmacologic measures versus pharmacologic ones demonstrated a significant decrease in narcotic use when nonpharmacologic agents were used with nonopioids. Based on that review, the team selected the following interventions: (1) application of ice packs applied to incisions in the postanesthesia care unit, (2) pain assessment within 30 minutes upon arrival to the ward, and (3) utilization of a specific postoperative appendectomy order set using alternating acetaminophen and ibuprofen with oxycodone available for rescue therapy. To validate that pain was controlled on return to the general unit, pain scores were documented within 30 minutes of arrival. The nurses documented the pain score using Verbal (numerical rating scale using 1 to 10) [16]; the Faces Pain Scale [17]; and the Face, Legs, Activity, Cry, Consolability [18] systems. If the patient remained asleep during the first 30 minutes, it was documented on the chart. All surgical providers including resident trainees were instructed to use the postoperative acute appendicitis order set in the electronic medical record. Directed feedback was provided to each surgical provider to encourage the use of the order set including electronic medical record built to direct the surgical provider to use the correct postoperative order set. Each patient was reviewed to determine if nonopioids were administered as ordered. To promote a cultural change and provide consistent messaging for all end users (surgical providers, trainees, nurses) and families, a stepping stones document was created and implemented.

Of note, intraoperative analgesia included injection of local anesthesia around incisions as well as administration of IV ketorolac prior to awakening the patient. These intraoperative processes were continued and not part of the QI process.

All of the outcomes were evaluated and discussed at project meetings and the department surgical educational series, ensuring appropriate use and monitoring of pain with weekly feedback to all end users for appendectomy patients.

## Plan–Do–Study–Act

All acute appendicitis patients undergoing laparoscopic appendectomy were included in the PDSAs. PDSA worksheets were completed for each change. End-user education was undertaken by a member of the project team. The changes were implemented from September 2017 to December 2017, and variables from each patient during this period were entered in the database. Weekly graphical feedback was presented on project boards with directed feedback to each end user. After the first week, adjustments were made to improve the workflow and ensure end-user buy in. Frequent sponsor engagement was used to keep all members of the team engaged with the process to monitor and communicate feedback to all end users. Sponsors were members of the executive team with authority who acted as liaisons with leadership to meet strategic aims, provide resources, help overcome any barriers, and hold the QI team accountable.

Outcome variables for the study included pain scores, inpatient postoperative IV and enteral narcotics use, and number of opioid doses prescribed at discharge. Other variables included length of stay, returns to system for pain or constipation, and total doses of opioids prescribed at discharge. Trends were followed using run charts to assess for special cause variation and sustainment of changes. Excluded patients included transfers from another facility postoperatively, incidental appendectomies, and chronic abdominal pain. Outliers that were not included were nonoperative management failure with subsequent appendectomy (*n* = 3), complex social situation (*n* = 1), and patients with complex clinical comorbidities (*n* = 3) who required customized pain management.

## Statistical Methods

Control P charts were used to trend the baseline and intervention data for returns to the system (visits to the emergency room, clinic, or readmission to medical center), ice pack placement in the postanesthesia unit, use of appendectomy order set, and opioid administration postoperatively and to assess for sustainment or special cause variation. The pre- and postimplementation groups were compared using the Wilcoxon rank sum test for continuous characteristics and the *χ*^2^ test for categorical variables. The *t* test was used to determine if there was a significant difference between the pre- and postimplementation groups. The exact test was used to demonstrate the relationship between opioids prescribed at discharge and having a return for pain or constipation.

## Results

**Fig 2:**
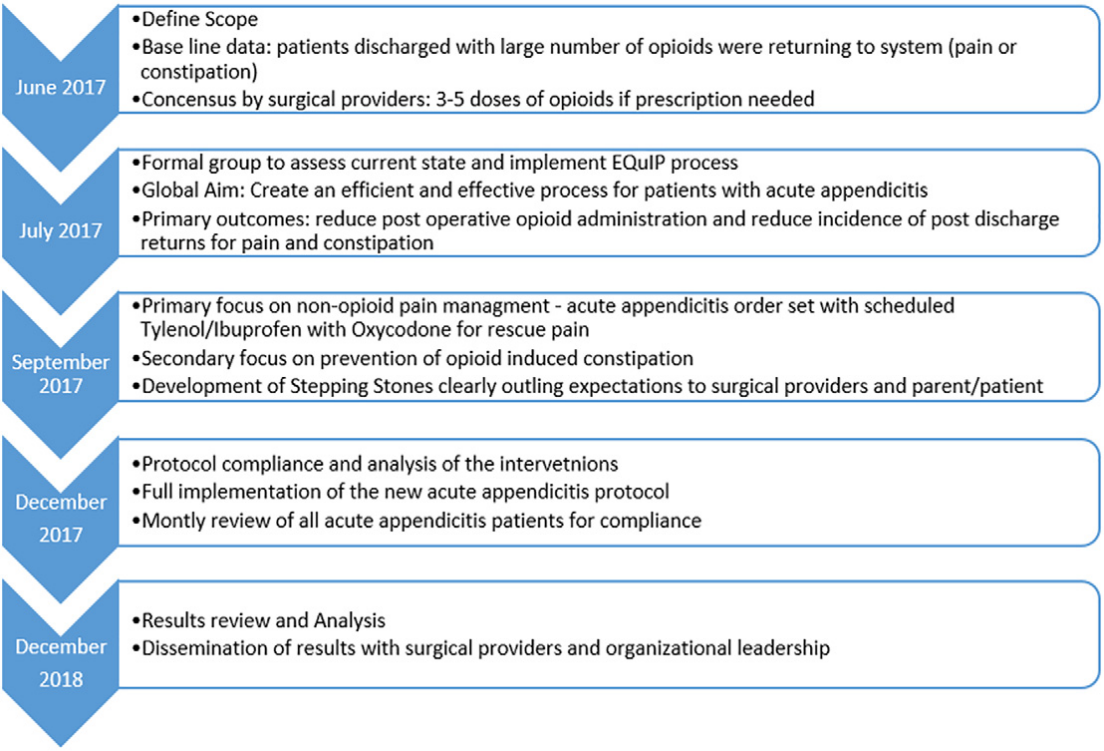
Flowchart showing timeline of development and implementation of opioid reduction quality improvement protocol for acute appendicitis patients.

The timeline and implementation of the quality improvement initiative are outlined in Figure 2. It took approximately 3 months after the initial meetings to establish the interventions and present to surgical leadership for implementation.

### Demographic Characteristics

Table 1 compares the pre- and postimplementation groups. There were no statistically significant differences in age or sex. There were 814 patients in the preimplementation group and 263 patients in the postimplementation group. Overall, the study populations were similar with the same surgical provider team during the 2 time periods.

**Table 1:** Comparison of preimplementation groups with demographics and outcome variables

	Preimplementation 2013–2016	Postimplementation 9/2017–12/2018	*P* value
No. of patients with laparoscopic appendectomy for acute appendicitis	814	263	NA
Male (%)	478 (58.7%)	154 (58.6%)	.962⁎
Median age in years (range) Mean ± SD	12.3 (1.0–20.3) 12.3 ± 3.6	12.2 (3.7–18.4) 12.1 ± 3.5	.420‡
Median total IV postop opioid doses (range) Mean ± SD	0 (0–7) 0.37 ± 0.90	0 (0–0) 0.0 0.0	<.001‡
Median total enteral postop doses (range) Mean ± SD	2 (0–14) 2.7 ± 1.8	0 (0–4) 0.7 ± 0.9	< .001‡
Patients with opioid prescription at discharge (%)	793 (97.4)	176 (66.9)	< .001†
Median no. of opioid doses prescribed (range) Mean ± SD	17 (2–139) 17.6 ± 9.9	5 (2–20) 5.2 ± 3.1	< .001‡
Opioid prescriptions filled (ePDMP review) (%)	474 (59.8)	110 (62.5)	.5514†
No. of patients with return for pain	Readmission: 3 Unscheduled clinic visit: 1 Return to ED: 6 Total: 10	Readmission: 1 Unscheduled clinic visit: 1 Return to ED: 0 Total: 2	.7407†
No. of patients with return for constipation	Readmission: 5 Unscheduled clinic visit: 1 Return to ED: 15 Total: 21	Readmission: 3 Unscheduled clinic visit: 1 Return to ED: 1 Total: 5	.6485†
Median length of stay in hours (range) Mean ± SD	21.9 (2.0–95.6) 22.9 ± 10.7	17.2 (1.1–68.5) 18.5 ± 10.6	< .001‡

Comparison of preimplementation and postimplementation groups with demographics. Statistical significance was demonstrated for parenteral and enteral opioid use postoperatively, prescriptions provided and filled at discharge, total doses of opioids prescribed, and length of stay. *T*, *t*-test; *C*, *χ*^2^; *W*, Wilcoxon rank sum test; *+*, exact test.

*ePDMP*, Enhanced Prescription Drug Monitoring Program.

⁎ *χ*^2^ test.

† Fisher exact test.

‡ Wilcoxon rank sum test.

### Compliance

Postimplementation, 66.9% had local ice packs in recovery unit, 87.5% had appropriately documented pain scores on return to the unit, and the order set was used in 94.7%.

### Opioid Administration

**Fig 3:**
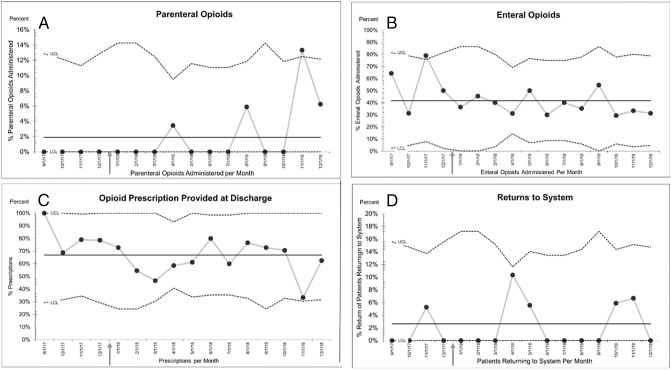
Controls charts specifying upper and lower control limits for assessing variability in conforming to the QI process. The center line is the expected value of proportions based on the sample size. The arrow denotes the point when the protocol was fully implemented (September 2017 to December 2018). The sample size for the control chart is consistent with the upper and lower limits of the distribution with a similar sample size. Controls charts A-D demonstrate consistent practice for the QI process with no special cause variation determined. A, Utilization for the postimplementation period of parenteral opioids. B, Utilization of enteral opioids. C, Discharge opioids for the postimplementation. D, Returns to health care system P chart for assessment of outcomes.

Parenteral opioids were administered in 22.5% of patients during the preimplementation period, whereas only 1.9% of the postimplementation group received parenteral opioids (<.001^W^) (Fig 3, *A*). Enteral opioids were administered in 92.4% of the preimplementation group, whereas only 41.8% of the postimplementation group had administration of enteral opioids (< .001^W^) (Fig 3, *B*).

Prior to implementation of the QI project, 97.4% (59.8% filled) of appendectomy patients received opioid prescriptions, whereas only 66.9% (62.5% filled) were prescribed opioids postimplementation (< .001^W^) (Fig 3, *C*). There was a statistically significant change in the number of opioid doses prescribed at discharge from the preimplementation period to the postimplementation period (< .001^W^). The number of prescriptions filled at discharge is not statistically significant.

Both the preimplementation group and postimplementation group were placed on a bowel regimen postoperatively, which was continued after discharge if clinically indicated. Prior to implementing the QI process, returns to the system for pain were 10.7% and constipation was 31.3%. After implementation, returns to the system decreased for pain (0.76%) and constipation (1.9%). These changes are not statistically significant (Fig 3, *D*).

## Discussion

Appendicitis is the most common surgical emergency in children [19]. Therefore, because appendectomy is often the index exposure for opioid administration in these patients, greater attention to postoperative opioid utilization is warranted. In a retrospective review, Anderson et al demonstrated a variation in opioid prescribing and the complications associated with opioid exposure postoperatively in patients undergoing appendectomy for appendicitis [20]. Given significant variation in perioperative analgesic practices, along with the implications of narcotic use at both the patient and institutional levels, this QI initiative was created to reduce opioid utilization in the perioperative period as well as reduce the overall returns to the system from narcotic-related complaints.

Characterizing historical perioperative pain management was essential in creating the aims for our study. It became quickly apparent that there was wide variation among our providers in the management of postoperative pain and in opioid prescribing and administration practices in response to perceived characterization of pain. By targeting these specific areas, we were able to show a statistically significant improvement in both enteral and parenteral opioid administration and enteral opioid prescriptions provided at discharge. These changes were made possible through targeted standardization of the postprocedural workflow that included a reliable way to measure pain scores, placement of ice packs immediately after appendectomy, and use of standardized postoperative order set. An essential factor in sustaining our program was providing directed feedback to surgeons to improve compliance with the postoperative order set, as well as education of both the patients and parents using the stepping stone document to appropriately adjust expectations. Compliance with our new bundle was ensured by reviewing adherence to the protocol through a structured review process and providing real-time feedback—both positive and negative—to the responsible parties with compliance or deviation from the protocol. One factor that diminished compliance, especially with ice pack placement, was the variation in nursing staff coverage on holidays and weekends. Knowledge of the process was decreased when there was staff only working in a casual position (less than a full-time equivalent or cross-coverage from other areas of the operating room). This study illustrated that the use of standardization decreases variation in both provider and nursing practice.

Utilization of quality tools can allow for consistency in the improvement process. As an academic training center, an essential part of maintaining these changes includes ongoing education of the guideline and order set to new trainees every 6 weeks. Furthermore, creating and implementing change require a shift in culture and buy-in from all members of the team. The way that we did this was to include nurses, providers, resident trainees, and staff at all levels in the process to provide perspective and feedback on how to structure these changes in a thoughtful and meaningful way. We also scheduled frequent meetings with established expectations and “homework” (creating stepping stones document; follow-up with end users for feedback on what was working and not working; barriers; and ongoing education on project boards to nurses, providers, and trainees) to encourage team member participation and hold all team members accountable. To create sustainable changes, we had to target specific objectives and limit the scope of our interventions so that we were not working beyond the capabilities of the system. However, one of the greatest benefits is we can now not only implement this process to further streamline our care for appendicitis patients but use this infrastructure to create other similar quality initiatives.

Limitations of this study include those inherent with a single-institution study. Although patients were not compared in a randomized manner, we did use a comparative control group with similar baseline demographics. The data collection and sharing may have triggered a Hawthorne (or observer) effect, whereby the desired outcome was a temporary change that would disappear once the end users were no longer being observed. Additionally, opioid prescription practices are different in each state based on their legislature, so our results may not be applicable to other centers. Also, other centers may have different opioids on formulary or different first-line pain medications due to insurance coverage; hence, they may use a different regimen. However, regardless of the agent, the overall utilization was decreased. Also, we had a very specific regimen, but we did not account for patients that received regional analgesia nerve blocks in lieu of infiltration into the incision.

In conclusion, targeted multidisciplinary groups can be used to evaluate a problem and to recommend and implement change. This initiative was successful in decreasing perioperative opioid utilization and returns to the system by providing an infrastructure that allows for decreased variability, improved compliance, and better outcomes in patients undergoing surgery for pediatric acute appendicitis.

## Author Contribution

QI team members and Daniel Eastwood, MCW Biostatistician.

There are no known conflicts of interest associated with this work. All authors made substantial contributions to the design, analysis, and interpretation of data for the work. All authors worked on drafting or revising the manuscript, and gave final approval of the version to be published. All authors are accountable for the work with regard to its accuracy and integrity.

## Conflict of Interest

There are no conflicts of interest by any of the authors.

## Funding Sources

This research was not supported by grants or funding agencies in the public, commercial, or not-for-profit sectors.
